# Determination of Tympanostomy Tube Types for Otitis Media with Effusion in Patients with Cleft Palate: Comparison between Paparella Type 1 and Type 2 Tubes

**DOI:** 10.3390/jcm12206651

**Published:** 2023-10-20

**Authors:** Jungho Ha, Ga Young Gu, Se Hyun Yeou, Hantai Kim, Oak-Sung Choo, Jeong Hun Jang, Hun Yi Park, Yun-Hoon Choung

**Affiliations:** 1 Department of Otolaryngology, Ajou University School of Medicine, Suwon 16499, Republic of Korea; jhflyflyfly@ajou.ac.kr (J.H.); sesoo825@naver.com (G.Y.G.); scieth17@naver.com (S.H.Y.); jhj@ajou.ac.kr (J.H.J.); hunyi@ajou.ac.kr (H.Y.P.); 2 Department of Medical Sciences, Ajou University Graduate School of Medicine, Suwon 16499, Republic of Korea; noto.hantai@gmail.com (H.K.); oschoo1202@gmail.com (O.-S.C.); 3 Department of Otorhinolaryngology, Konyang University College of Medicine, Daejeon 35365, Republic of Korea; 4 Department of Otorhinolaryngology-Head and Neck Surgery, Kangnam Sacred Heart Hospital, Hallym University College of Medicine, Seoul 07441, Republic of Korea

**Keywords:** tympanostomy tube, ventilation tube, otitis media with effusion, cleft palate, palatoplasty

## Abstract

This study examined the effects of different types of tympanostomy tubes in pediatric patients undergoing cleft palate (CP) surgery in order to provide guidance for the proper insertion of tympanostomy tubes in the management of otitis media with effusion (OME). A total of 101 ears with middle ear effusion in 51 patients with CP were included in this study. Patients underwent palatoplasty and tympanostomy tube surgery at the same time. The type of tube inserted (Paparella type 1 or 2), the severity of CP, and types of palatoplasty surgeries were investigated. All patients were followed up for at least 6 months, and recurrence rates, complications, and reinsertion surgery were evaluated. The rate of OME recurrence after spontaneous tube extrusion was significantly higher in the type 1 group than in the type 2 group (44.3% vs. 19.4%, respectively, *p* = 0.016). Persistent eardrum perforation was more common in the type 2 group than in the type 1 group (41.9% vs. 12.9%, respectively, *p* = 0.001). The tube reinsertion rate was higher in the type 1 group than in the type 2 group (22.9% vs. 3.2%, respectively, *p* = 0.015). The tube reinsertion rate decreased to 8.6% in cases of palatoplasty with Sommerlad’s technique, even with type 1 tube insertion, which was not significantly different from the reinsertion rate in the type 2 group (3.7%, *p* = 0.439). The Paparella type 1 tube would be a better choice in cases of palatoplasty performed using Sommerlad’s technique, particularly considering the higher rate of persistent eardrum perforation after extrusion associated with the Paparella type 2 tube. Alternatively, a larger size type 2 tube may be considered in other surgeries to decrease the frequency of recurrence and tube reinsertion.

## 1. Introduction

Cleft lip and cleft palate (CP) are openings or splits in the upper lip and palate, respectively [1]. The tensor veli palatini muscle and levator veli palatini muscle in the soft palate do not function properly in children with CP, which makes them unable to open the Eustachian tube. This functional obstruction causes otitis media with effusion (OME) in children with CP, which compromises proper Eustachian tube opening. OME exhibits a high incidence rate in pediatric patients with CP [2,3,4,5].

Untreated OME may result not only in hearing loss but also in long-term impairments of speech, language, and cognitive development. Furthermore, it can result in structural lesions of the tympanic membrane (TM) or middle ear clefts, such as retraction pockets, atelectasis, or cholesteatoma [3,5,6,7]. Therefore, surgical management, such as tympanostomy tube surgery (TTS), may be required to prevent these developmental problems [6,7]. If necessary, TTS is usually performed during the first 5 years of life [8]. There are several types of tubes, including Armstrong, T-tubes, and Paparella tubes [9]. Two types of Paparella tubes may be used, i.e., the Paparella type 1 ventilation tube with an inner diameter of 1.14 mm and the Paparella type 2 ventilation tube with a larger inner diameter of 1.52 mm. In previous studies, the removal of asymptomatically retained tympanostomy tubes was recommended after 18 months, but they may also remain in place for 4–5 years [9,10]. During this period, because the type 2 tube has a larger vent, it can provide better middle ear aeration. However, it is also associated with a higher risk of eardrum perforation after extrusion of the tube.

While there is no doubt about the need for TTS in CP patients with OME, there is no consensus on the most appropriate size of the tube. Several factors, such as the classification of the defect or surgical technique, may affect the function of the Eustachian tube in patients with CP [4,11]. Therefore, the most appropriate size of the tube should be determined differently from TTS in patients without such defects. This study reviewed the outcomes of TTS in patients with CP to determine the most appropriate size of the tube.

## 2. Materials and Methods

### 2.1. Study Population

Fifty-one pediatric patients with CP who underwent both palatoplasty and TTS between 2005 and 2014 were included in the study. Both surgeries were performed simultaneously at Ajou University Hospital (Suwon, Republic of Korea). Patients were followed up for at least 6 months. We recorded patients’ ages, types of CP surgery, types of ventilation tubes, postoperative complications, and recurrence rates. All procedures were approved by the Institutional Review Board (Approval No. AJIRB-MED-MDB-21-706) and were performed in concordance with the 1964 Declaration of Helsinki and its later amendments.

### 2.2. Classification of CP

Types of CP were classified using the Veau classification system, which divides clefts into four groups according to the severity of palate involvement: Veau I, clefts are limited to the soft palate only; Veau II, involvement of the hard and soft palates but not alveolar structures; Veau III, involvement of hard and soft palates and alveolus unilaterally; and Veau IV, bilateral complete clefts involving soft and hard palates and alveolus bilaterally [12]. Sommerlad’s technique (Veau I and II), double opposing Z-plasty (Veau I), one-flap palatoplasty (Veau II–IV), and two-flap palatoplasty (Veau II–IV) were used for palatoplasty. Additional surgical techniques, either intravelar veloplasty or buccal fat pad flap, were applied if necessary for closure. Sommerlad’s technique is utilized for the primary closure of the cleft palate and involves extending the midline incision onto the posterior hard palate with elevation of a posterior mucoperiosteal flap to expose the hard palate’s posterior border and radical muscle dissection without the use of relaxing incisions. Additionally, it may or may not include intravelar veloplasty. The technique entails dissecting anteriorly malpositioned bundles, including the levator veli palatini and a small part of the tensor veli palatini muscles, from the posterior edge of the hard palate, followed by repositioning them to the most posterior part of the soft palate [11].

### 2.3. Tympanostomy Tube Surgery

Before performing palatoplasty, experienced otologists evaluated the tympanic membrane of the patients. If either middle ear effusion or TM retraction was detected, TTS was performed simultaneously with palatoplasty. After palatoplasty under general anesthesia, TTS was performed under a microscope. One of the two types of ventilation tubes, Paparella type 1 or 2 (Medtronics Xomed Inc., Jacksonville, FL, USA), was consistently inserted in the anterior inferior quadrant (AIQ), except in a minority of cases with retracted or fragile TMs. Paparella type 2 ventilation tubes were primarily used for patients with CP until 2009.

### 2.4. Statistical Analysis

The χ^2^ test was used to assess differences among categorical data, and the independent *t*-test was used for numerical data with an approximately normal distribution. All statistical analyses were performed using IBM SPSS Statistics for Windows (Version 25.0, 21.0; Armonk, NY, USA). In all analyses, a *p*-value < 0.05 was taken to indicate statistical significance.

## 3. Results

All subjects underwent both CP surgery and TTS at 1.8 years old (22.1 ± 25.7 months). Overall, 23 patients (45.1%) were male and 28 (54.9%) were female. Only one patient received unilateral insertion. A total of 101 ears were analyzed.

### 3.1. Characteristics of Patients According to the Type of Tube Inserted

Of 101 ears, Paparella type 1 tubes were inserted in 70 ears, and Paparella type 2 tubes were inserted in the other 31 ears. Of the 55 ears that underwent surgery prior to 2010, Paparella type 2 ventilation tubes were inserted in 27 ears (49.1%), while Paparella type 1 ventilation tubes were used in 28 ears (50.9%). However, from 2010, Paparella type 1 ventilation tubes were more commonly used, with 42 ears (91.3%) receiving type 1 tubes and only 4 ears (8.7%) receiving type 2 tubes.

The average ages at the time of the operation were 21.7 ± 25.9 months in the type 1 group and 12.0 ± 5.8 months in the type 2 group (*p* = 0.004). There were no differences in the sex ratio between the groups. Veau II was the most common type of CP (61.4% in the type 1 group and 77.0% in the type 2 group), and there was no association between Veau classification and inserted tube size (*p* = 0.227). While most of the patients in the type 2 group underwent palatoplasty using Sommerlad’s technique (87.1%), not only the Sommerlad’s technique (52.9%) but also one-flap (14.3%) and two-flap (31.4%) palatoplasties, were performed in the type 1 group. Additional surgeries, such as intravelar veloplasty or buccal fat pad flap, were performed mainly in the type 1 group (28.6% and 37.1% of patients in the type 1 group, respectively). The Paparella type 2 tube could remain in the eardrum for a longer period than in the type 1 tube (42.3 ± 22.2 months vs. 15.5 ± 14.0 months, respectively, *p* < 0.001). The mean follow-up period for patients was significantly longer in the type 2 tube group, 50.4 ± 28.8 months, compared to the type 1 tube group, 30.5 ± 23.9 months (*p* < 0.001) (Table 1).

### 3.2. Recurrence and Persistent Eardrum Perforation after Tube Extrusion

**Figure 1:**
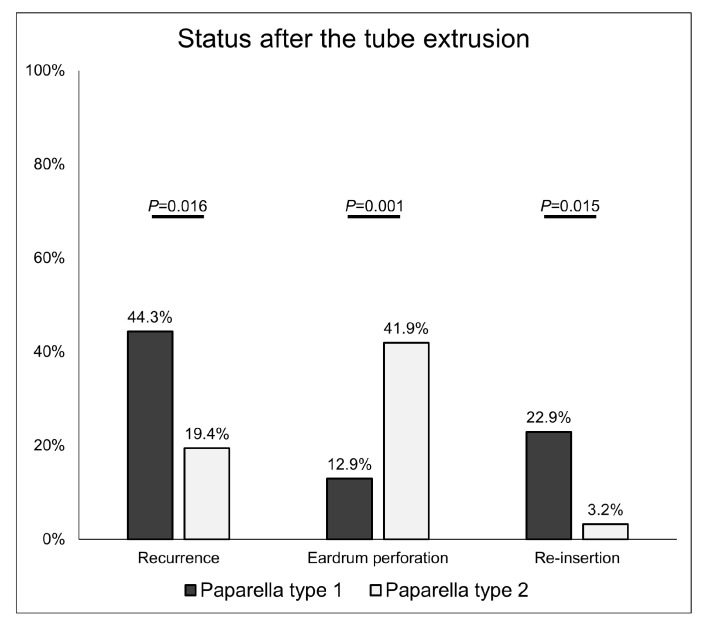
Recurrence and eardrum perforation rates according to the type of tympanostomy tube after tube extrusion. If neither effusion nor retraction resolved spontaneously after the recurrence, the tube was reinserted.

Tubes were spontaneously extruded from the eardrum. The risk of recurrence was higher in the type 1 group than in the type 2 group (44.3% vs. 19.4%, respectively, *p* = 0.016). The rate of persistent eardrum perforation after tube extrusion was significantly higher in the type 2 group than in the type 1 group (41.9% vs. 12.9%, respectively, *p* = 0.001). This result might be caused by the larger diameter of type 2 tubes and the extended maintenance period. However, in most patients, despite the recurrence of effusion or retraction, they recovered spontaneously. The rates of tube reinsertion were 22.9% in the type 1 group and 3.2% in the type 2 group (*p* = 0.015) (Figure 1).

### 3.3. Factors Affecting Recurrence and Tube Reinsertion Surgery for Tube Reinsertion in Type 1 Tympanostomy Tube Patients

**Figure 2:**
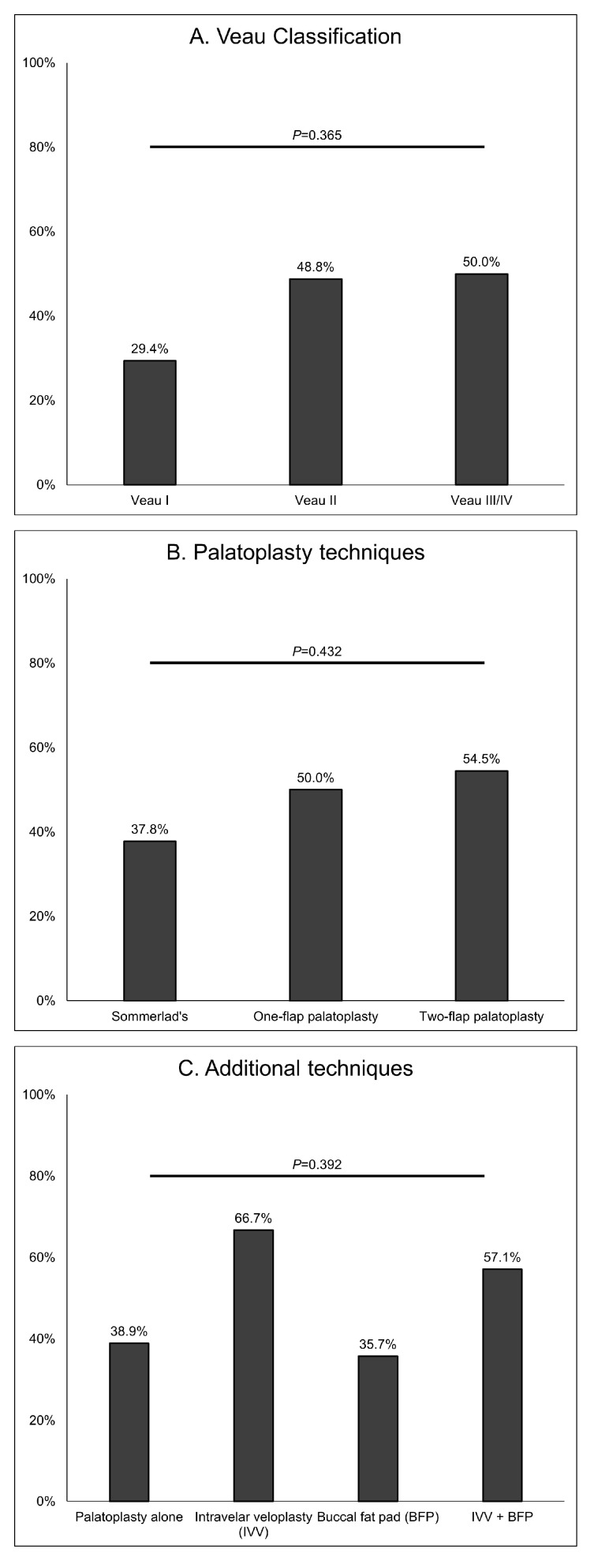
Recurrence rates in patients undergoing type 1 tympanostomy tube insertion according to Veau classification, palatoplasty technique, and additional techniques with palatoplasty. Double opposing Z-plasty was performed in only one patient and so was not included in the analysis.

We investigated the recurrence and tube reinsertion rates according to the severity of CP (Veau classification) and the types of palatoplasty surgical technique in patients who received TTS with the Paparella type 1 tube. The recurrence rates were higher in cases of Veau II–IV than in Veau I (29.4%, 48.8%, and 50.0% in Veau I, II, and III/IV, respectively), but the difference was not statistically significant (*p* = 0.365). The recurrence rate was lower when Sommerlad’s technique was used for palatoplasty (37.8%) compared to other methods (50.0% for one-flap palatoplasty and 54.5% for two-flap palatoplasty), but these differences were also not significant (*p* = 0.432). The recurrence rates tended to be higher in cases in which intravelar veloplasty was performed in addition to palatoplasty (66.7% for intravelar veloplasty and 57.1% for intravelar veloplasty + buccal fat pad) (Figure 2).

**Figure 3:**
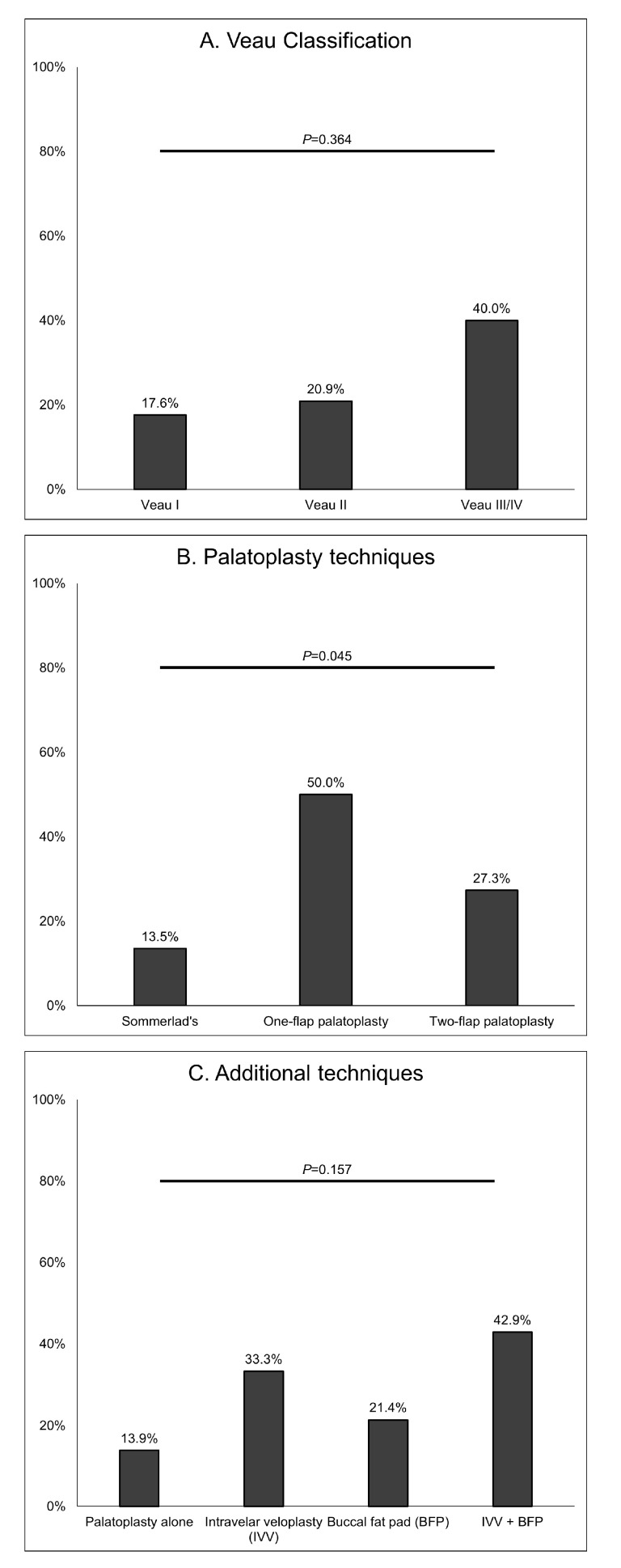
Reinsertion rates in patients undergoing type 1 tympanostomy tube insertion according to Veau classification, palatoplasty technique, and additional techniques with palatoplasty. Double opposing Z-plasty was performed in only one patient and so was not included in the analysis.

We also investigated the reinsertion rates according to Veau classification and surgical technique in the type 1 group. Veau III/IV and intravelar veloplasty cases tended to show higher reinsertion rates (40.0% for Veau III/IV, 33.3% for intravelar veloplasty, and 42.9% for intravelar veloplasty + buccal fat pad). Recurrence rates showed a similar pattern. However, the reinsertion rate was significantly lower when Sommerlad’s technique was performed (13.5%, *p* = 0.045) (Figure 3).

### 3.4. Recurrence and Tube Reinsertion Rates in Patients Treated Using Sommerlad Palatoplasty without Intravelar Veloplasty

**Figure 4:**
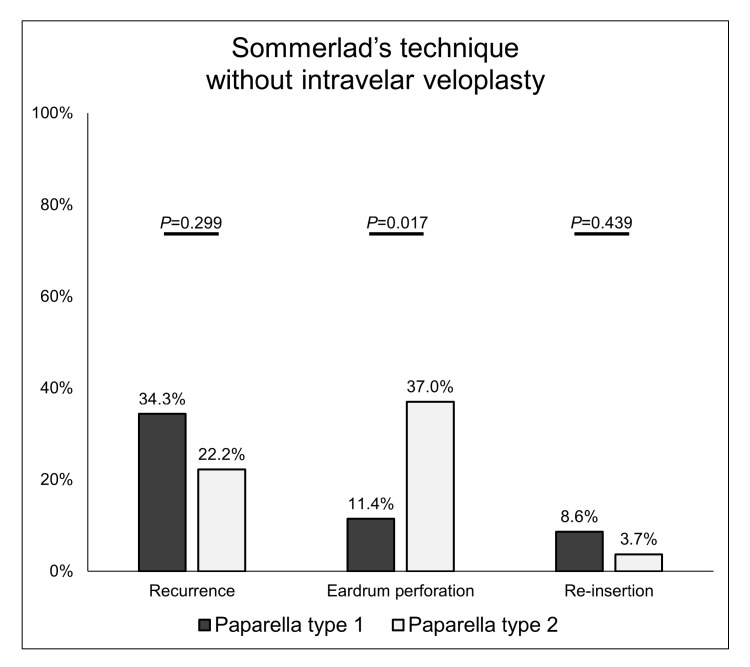
Recurrence and eardrum perforation rates according to the type of tympanostomy tube insertion in patients undergoing palatoplasty using Sommerlad’s technique without intravelar veloplasty.

Sommerlad’s technique can include or exclude intravelar veloplasty. Taken together, our observations showed that Sommerlad’s technique could lower the risk of recurrence and the reinsertion rate, while intravelar veloplasty tended to increase them (Figure 2 and Figure 3). Therefore, the recurrence, eardrum perforation, and reinsertion rates after tube extrusion were analyzed only in patients who underwent palatoplasty using Sommerlad palatoplasty without intravelar veloplasty. There were no significant differences between the type 1 and type 2 groups in recurrence rate (34.3% vs. 22.2%, respectively, *p* = 0.299) or reinsertion rate (8.6% vs. 3.7%, respectively, *p* = 0.439). However, the rate of persistent eardrum perforation was significantly higher in the type 2 group than in the type 1 group (37.0% vs. 11.4%, respectively, *p* = 0.017) (Figure 4).

## 4. Discussion

More than 80% of patients with CP develop OME within the first 24 months of life [5,13,14]. Middle ear effusion negatively affects not only their hearing but also their linguistic and learning abilities [6,7,15]. Additionally, it can lead to structural abnormalities in the TM or middle ear cleft, such as retraction pockets, atelectasis, or cholesteatoma [3,5,6,7]. Although this negative effect is not clear [16,17], TTS has often been performed routinely with palatoplasty as tube insertion is not an aggressive procedure, and general anesthesia is applied for palatoplasty.

However, there has been some debate regarding the most appropriate tube size. Small Paparella type 1, or short-term tubes, are associated with a risk of recurrence and relatively short indwelling time, while large Paparella type 2, or long-term tubes, are associated with persistent eardrum perforation [18,19,20]. These patterns were also observed in CP patients. In this study, 44.3% of patients in the type 1 group showed recurrence of effusion or retraction after tube extrusion and 22.9% underwent reinsertion of the tube because the middle ear lesion did not resolve spontaneously. On the other hand, in the type 2 group, the recurrence rate was 19.4%, and the reinsertion rate was only 3.2%. However, persistent eardrum perforation occurred in 41.9% of patients in the type 2 group. The presence of such perforation is probable to have contributed to a reduction in the recurrence of effusion through ventilation of the middle ear space. As each type of tympanostomy tube has clear advantages and disadvantages, it is difficult to determine the most appropriate tube size for insertion in CP patients.

Our results suggest that the Paparella type 1 tube may be a better choice when applying Sommerlad’s technique. There were no significant differences between the type 1 and 2 groups in the recurrence or tube reinsertion rates, but the rate of eardrum perforation was higher in the type 2 group. Therefore, there is no reason to insert the larger tube in CP patients undergoing palatoplasty using Sommerlad’s technique. Two studies have shown that Sommerlad’s technique is more advantageous in CP patients with OME. Hogoughi et al. [11] reported that middle ear effusion could improve without TTS in cases undergoing palatoplasty with intravelar veloplasty using Sommerlad’s technique. In addition, D’Andréa et al. [21] reported that implementing this method early for cases with intravelar veloplasty reduced persistent OME compared to Veau-Wardill-Kilner palatoplasty. Sommerlad’s technique involves palatal musculature reconstruction, which distinguishes it from non-anatomical reconstruction techniques, such as the Veau-Wardill-Kilner procedure. Palatal musculature reconstruction is more advantageous in the improvement of Eustachian tube function, thus helping with the resolution of OME [13,22,23,24]. Our results showing that Sommerlad’s technique had a positive effect on the course of OME are consistent with previous reports. However, unlike previous studies, it is difficult to discuss the effects of intravelar veloplasty as only one patient in our study population underwent intravelar veloplasty together with Sommerlad’s technique, and effusion recurred in both ears of this patient after tube extrusion. The inclusion of this patient’s data in the analysis did not significantly alter the results (Supplemental Figure S1). Consequently, taken together with the results of previous studies, we suggest that the type 1 tube should be chosen for CP patients undergoing palatoplasty using Sommerlad’s technique with intravelar veloplasty [11,21].

In general, a more severe Veau classification was associated with higher recurrence and reinsertion rates, but these relations were not significant. We believe that it is more important to determine the tube size by considering the palatoplasty surgical technique rather than the severity of CP itself. Even in relatively mild cases, the function of the Eustachian tube may not sufficiently recover if the surgery cannot successfully reconstruct the palatal musculature. Therefore, we recommend that palatoplasty be performed before TTS. When performing TTS, the surgeon should identify the surgical method used for palatoplasty and then insert a type 1 tube when Sommerlad’s technique is used, and otherwise consider type 2 tube insertion. In this study, the follow-up period to investigate the reinsertion rate was approximately 50 months for the type 2 tube and 30 months for the type 1 tube. Although these periods were relatively long, the observation period may not have been sufficient considering the long maintenance period of the type 2 tube. Therefore, it is possible that the differences in the reinsertion rate according to the surgery were also found in the type 2 tube group. This may be the limitation of the study.

## 5. Conclusions

The size of the tympanostomy tube should be determined by considering the surgical method used for palatoplasty in TTS performed simultaneously. In cases in which palatal musculature reconstruction is performed with Sommerlad’s technique, the Paparella type 1 tube would be a better choice than the type 2 tube. Alternatively, a larger size type 2 tube may be considered in other surgeries to decrease the frequency of recurrence and tube reinsertion.

## Figures and Tables

**Table 1:** Characteristics of patients according to the types of the inserted tube.

	Paparella Type 1 (*n* = 70)	Paparella Type 2 (*n* = 31)	*p*-Value
Operation age (months) ^a^	21.7 (25.9)	12.0 (5.8)	0.004 *
Gender			0.931 **
Male	30 (42.9%)	13 (41.9%)	
Female	40 (57.1%)	18 (58.1%)	
The Veau classification			0.227 **
Veau I	17 (24.3%)	9 (9.7%)	
Veau II	43 (61.4%)	22 (77.0%)	
Veau III/IV	10 (14.3%)	6 (19.4%)	
Palatoplasty techniques			
Sommerlad’s technique	37 (52.9%)	27 (87.1%)	0.001 **
Double opposing Z-plasty	1 (1.4%)	1 (3.2%)	0.550 **
One-flap palatoplasty	10 (14.3%)	1 (3.2%)	0.100 **
Two-flap palatoplasty	22 (31.4%)	2 (6.5%)	0.007 **
Additional techniques			
Intravelar veloplasty	20 (28.6%)	0 (0.0%)	0.001 **
Buccal fat pad flap	26 (37.1%)	1 (3.2%)	<0.001 **
Duration of tube maintenance (months) ^a^	15.5 (14.0)	42.3 (22.2)	<0.001 *
Follow-up period (months)	30.5 (23.9)	50.4 (28.8)	<0.001 *

^a^ Values are presented as mean (SD); * *p*-value from the independent *t*-test; ** *p*-value from the *Chi*-squared test.

## Data Availability

The datasets generated and/or analyzed in the current study are available from the corresponding author upon reasonable request.

## Supplementary Materials

The following supporting information can be downloaded at: https://www.mdpi.com/article/10.3390/jcm12206651/s1, Supplemental Figure S1. Recurrence and eardrum perforation rates according to the type of tympanostomy tube insertion in patients undergoing palatoplasty using Sommerlad’s technique with or without intravelar veloplasty.
