# Supplementary material for: Determination of Tympanostomy Tube Types for Otitis Media with Effusion in Patients with Cleft Palate: Comparison between Paparella Type 1 and Type 2 Tubes
Source: J Clin Med. 2023 Oct 20;12(20):6651. doi: 10.3390/jcm12206651 (PMC10607012; doi:10.3390/jcm12206651)
Supplement: Supplementary file 1 [file jcm-12-06651-s001.zip › jcm-2630186-supplementary.pdf]

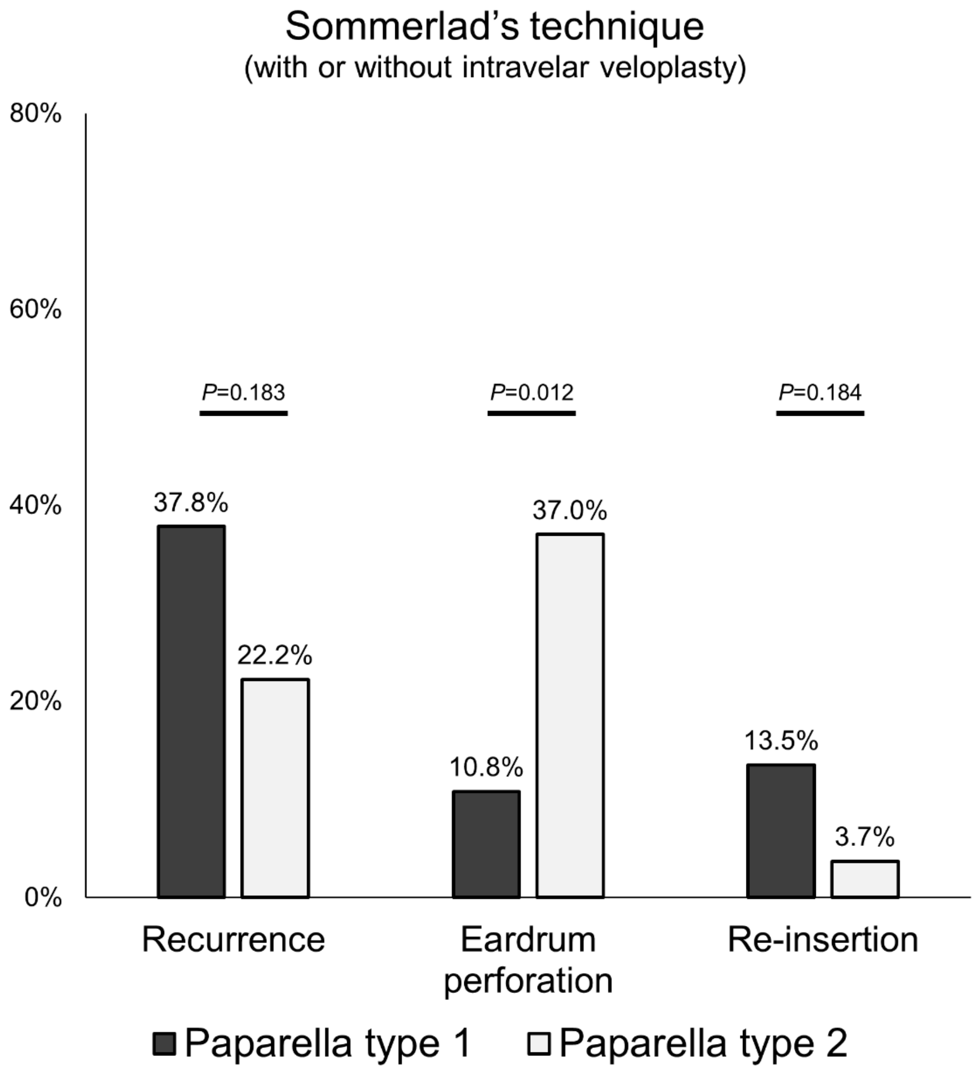

Supplemental Figure S1. Recurrence and eardrum perforation rates according to type of tympanostomy tube insertion in patients undergoing palatoplasty using Sommerlad's technique with or without intravelar veloplasty.
